# Neurocognitive poetics: methods and models for investigating the neuronal and cognitive-affective bases of literature reception

**DOI:** 10.3389/fnhum.2015.00186

**Published:** 2015-04-16

**Authors:** Arthur M. Jacobs

**Affiliations:** ^1^Department of Experimental and Neurocognitive Psychology, Freie Universität BerlinBerlin, Germany; ^2^Center for Cognitive Neuroscience (CCNB), Freie Universität BerlinBerlin, Germany; ^3^Dahlem Institute for Neuroimaging of Emotion (D.I.N.E.), Freie Universität BerlinBerlin, Germany

**Keywords:** neurocognitive poetics, fiction feeling hypothesis, Panksepp-Jakobson hypothesis, foregrounding, immersion, absorption, poetic function, neuroaesthetics, neuroliterature, emotion potential

## Abstract

A long tradition of research including classical rhetoric, esthetics and poetics theory, formalism and structuralism, as well as current perspectives in (neuro)cognitive poetics has investigated structural and functional aspects of literature reception. Despite a wealth of literature published in specialized journals like *Poetics*, however, still little is known about how the brain processes and creates literary and poetic texts. Still, such stimulus material might be suited better than other genres for demonstrating the complexities with which our brain constructs the world in and around us, because it unifies thought and language, music and imagery in a clear, manageable way, most often with play, pleasure, and emotion (Schrott and Jacobs, 2011). In this paper, I discuss methods and models for investigating the neuronal and cognitive-affective bases of literary reading together with pertinent results from studies on poetics, text processing, emotion, or neuroaesthetics, and outline current challenges and future perspectives.

*Aesthetic value, then, is like the wind—we know of its existence only through its effects (Iser, 1976)*.

## Neurocognitive Poetics: What for?

When Russians read Pushkin’s poem “Ja vas ljubil” (english translation: I loved you) or Germans Hölderlin’s “Hälfte des Lebens” (english translation: Half of life), their brains fulfill a miraculous process: they provide the neuronal bases of sounds and images, of more or less conscious feelings and thoughts emerging out of phonemes, syllables, words and word order, rhymes, rhythm, and more often than not, of readers’ subjective, self-rewarding experience of beauty and harmony. Researchers interested in poetics and literature reception in general can take advantage of the ever-changing neurocognitive methods to learn more about this miraculous process of creating pleasure and meaning out of formalized speech elements, but they face a number of theoretical and methodological challenges I would like to address here. Whoever listened to or read Dylan Thomas’ “Do Not Go Gentle Into That Good Night” has little doubt that the goose bumps accompanying the reception of poetry, that Emily Dickinson and Robert Graves have told us about, are true; the muscles relax, while the mind can focus; one is closer to laughter than to tears, inhales more deeply and a light feeling of ebriety spreads around. The french author Raymond Roussel compared this with a sober drunkenness and Coleridge with the effect of a few glasses of booze in a conversation (Schrott and Jacobs, 2011; p. 375). Turner and Pöeppel (1983) speak of “that pleasing sensation of fit and inevitability which is part of the delight of verse, and is so helpful to the memory”.

Despite these convincing testimonies of the beneficial effects of poetic language, entire libraries filled with books about it, its archaic origins and long-known usefulness for many practical purposes, we know only little about what happens in the brain when people read poetic or literary texts (cf. Ferstl, 2010; Mar, 2011). This stands in contrast to its undeniable general importance for religious, social, and economic rituals, or the process of education in nearly all literate traditions (Turner and Pöeppel, 1983). Forms of poetry are—often playfully—used to teach children to speak, read, write, or count, to bring sleep or consolation, or to sell books, music and other consumer products, or political ideas (Jacobs and Kinder, 2015). Moreover, metered poetry appears to be an ideal technique for stimulating and sensitizing the endogenous reward system of the brain, enabling enhancement of the integrative powers of our minds (Turner and Pöeppel, 1983). Finally, poetry might be well suited to compactly demonstrate the complexities with which our brains construct the world in and around us, unifying thought, language, music, and images with play, pleasure, and emotion. Poetic language plays with our affective and cognitive apparatus in a way that facilitates empirical investigation: it works with a catalog of formal stylistic devices and “figures of thought” (e.g., polysemy, irony, meiosis, oxymoron) which reflect partially innate perceptual, affective, and cognitive schemata and allow clear predictions about how (and where in the brain) such verbal stimuli are processed, for instance in analogy to stimuli producing visual illusions (Schrott and Jacobs, 2011), or basic emotions (Jacobs et al., 2015), thus “presenting to us an experience perfectly designed for the human brain” (Turner and Pöeppel, 1983).

Furthermore, poetry can generally be understood as inherently concerned with the expression and elicitation of affective meaning and emotions (Lüdtke et al., 2014) while being deeply rooted at the esthetical and perceptual level in the domains of speech and sound (Schrott and Jacobs, 2011). This becomes evident, for instance, by an emphasis of phonological units such as syllables or phonemes through diverse stylistic devices, like onomatopoeia or figures of self-similarity and parallelisms as rhyme, meter, or alliterations (Waugh, 1980). For example, in Goethe’s famous “Ein Gleiches” the onomatopoeic quality of the word “Hauch” (h, ch) is echoed in the rhyming sound (ch) and the juxtaposition of both generates a secondary affective meaning superimposed on the lexical meaning: “Your life, too, is like a breeze of wind and will pass away just as easily” (Neuhäuser, 1991; Aryani et al., 2015). These two major principles of the poetic genre, i.e., the prominence of sound properties and more or less subtly expressed or perceived affective meanings offer a wealth of research issues for a neurocognitive poetics perspective (Schrott and Jacobs, 2011).

Focusing on written texts, in this essay I discuss the state of the art of neurocognitive poetics, that is—in a broad sense—the transdisciplinary empirical investigation of and theorizing about (poetic) literature reception by eye or ear including its neuronal underpinnings, (cf. Jacobs, 2011). The inclusion of methods and models for investigating the neurocognitive processes associated with processing and experiencing literary texts is what basically distinguishes it from “Cognitive Poetics”, as pioneered by Tsur (1983, 1992; see also Stockwell, 2002, 2007),^1^ or from other seminal empirical and theoretical approaches to literature reception (e.g., Martindale, 1978; Schmidt, 1979, 1983; Van Dijk, 1979; Van Peer, 1986; Hoffstaedter, 1987; Miall, 1988, 1989, 1990; Zwaan, 1993; Miall and Kuiken, 1994; Oatley, 1994; Hanauer, 1997; Gerrig, 1998; Bortolussi and Dixon, 2003). The use of neurocognitive methods imposes, of course, certain constraints not met by “cognitive poetics” studies which might be objected on grounds of ecological validity or generality. I will discuss the risks and rewards of this methodological perspective later and start with an example of the potential rewards. Meanwhile it is worth noting that despite repeated critiques of neuroscientific perspectives on literature comprehension (e.g., Koepsell and Spoerhase, 2008) or interdisciplinary approaches to poetics in general (Sternberg, 2003), more than a few scholars from literature science recognize the potential benefits (Lauer, 2007; Gosetti-Ferencei, 2014; Salgaro, 2009; Lubrich et al., 2014). On the other hand, neuroscientists have repeatedly emphasized the benefits of studying literary language processing for understanding the workings of the mindbrain (e.g., Turner and Pöeppel, 1983; Mar, 2011; Schrott and Jacobs, 2011; Wallentin et al., 2011; Willems, 2015), both forming a nice match, as suitably expressed by Turner and Pöppel: “Poetry presents to the brain a system which is temporally and rhythmically hierarchical, as well as linguistically so, and therefore matched to the hierarchical organization of the brain itself”.

## The Fiction Feeling and Panksepp-Jakobson Hypotheses

**Figure 1:**
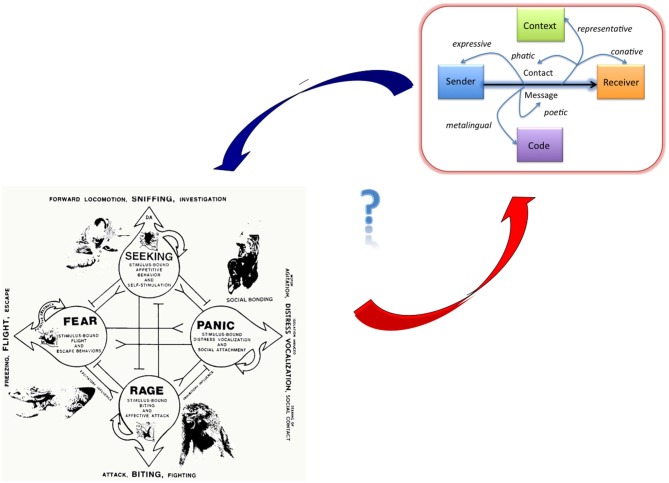
**Illustration of the Panksepp-Jakobson hypothesis linking neurobiological theories of emotion with complex linguistic models**. Bottom-left: four core affect systems (fear, rage, panic, and seeking; taken from Panksepp, 1998). Top-right: illustration of Jakobson’s extension of Bühler’s organon model of language describing the interplay between six language functions always operating in any communicative act in different mixtures.

Can neurocognitive poetics studies advance our understanding of how the mindbrain works when processing literature beyond what can be revealed by structural, theoretical, or behavioral studies alone? I think that an encouraging answer is given by the example of experimental tests of the *fiction feeling hypothesis* of literary reading, a key element of my neurocognitive poetics model (NCPM in short; Jacobs, 2011, 2015). Inspired by previous results indicating that children’s processing of stories eliciting affective and cognitive empathy is associated with medial and bilateral orbitofrontal cortex (OFC) activation (Brink et al., 2011), it states that narratives with emotional contents invite readers more to be empathic with the protagonists and immerse in the text world (e.g., by engaging the affective empathy network of the brain, mainly the anterior insula and mid-cingulate cortex), than do stories with neutral contents. The hypothesis is based on Kneepkens and Zwaan’s (1994) notion of *fiction emotions*, e.g., when readers experience fear as a consequence of events in the text world. To examine whether readers experience (vicarious) fear, happiness, or disgust, and account for potential differences in kind or degree with regard to real-life emotions, an additional hypothesis is needed that bridges the *language-emotion gap* (Panksepp, 2008; Schrott and Jacobs, 2011) and that is testable with methods allowing to measure affective responses independently of and supplementing explicit assessments, like verbal reports or rating scales.^2^ Such a hypothesis, termed the* Panksepp-Jakobson hypothesis* (Jacobs and Schrott, 2013; Jacobs et al., 2015) illustrated in Figure 1, states that since evolution had no time to invent a proper neuronal system for art reception, even less so for literary reading, the affective and esthetic processes we experience when reading (cf. Jakobson’s “poetic function”) must be linked to the ancient emotion circuits we share with all mammals, as perhaps best described by Panksepp (1998). Thus, when subjects experience and rate words or text passages as “fearful”, “disgusting”, or “beautiful”, neuronal networks systematically associated with fear and disgust (e.g., amygdala and insula), or reward and pleasure (e.g., ventral striatum, OFC) should be more active than in apppropriate (neutral) control conditions. To look for evidence for such neuronal activations which cannot be deliberately controlled represents a stronger test of the *fiction feeling hypothesis* than verbal reports alone, similar to bridging the *concept-action/emotion gaps* by testing the “embodied semantics” (Gallese and Lakoff, 2005) and “embodied emotions” (Niedenthal, 2007) hypotheses by using fMRI (e.g., Aziz-Zadeh et al., 2006; Nummenmaa et al., 2008).

Several recent neurocognitive studies on reading indeed provide support for both the *fiction feeling* and* Panksepp-Jakobson hypotheses*. For instance, Altmann et al. (2012) looked at whether readers’ affective mentalizing networks activated more likely in short stories with negative emotional contents than in stories with neutral valence. The results of a PPI analysis corroborated both hypotheses showing that with increasingly negative content, reading stories engaged the bilateral inferior frontal gyrus and additional subcortical structures commonly involved in emotion processing, such as the caudate body, or (left) amygdala. Additional evidence for the *Panksepp-Jakobson hypothesis* comes from a study using both surface and intracranial electroencephalography (EEG), showing that a brain region that responds to specific emotions in a variety of situations and stimuli (anterior insula) also selectively activates—as early as 200 ms post-stimulus—when sujects read disgusting words (Ponz et al., 2014). Further evidence in support of both hypotheses is discussed later.

## Methods and Tools for Neurocognitive Poetics

Readers’ responses to literary texts are determined by three groups of factors: text, context, and reader (i.e., skill, motivation, personality; cf. Dixon et al., 1993). Thus, a comprehensive neurocognitive poetics perspective should develop tools for describing all three relevant factors and their relative influences on the neuronal and mental processes underlying literary reading. Here, I focus on tools for text analysis, mentioning context and reader factors only peripherally (see Jacobs, 2011, 2015; for a more elaborate discussion of these).

## Text Analysis: A 4 × 4 Matrix

**Figure 2:**
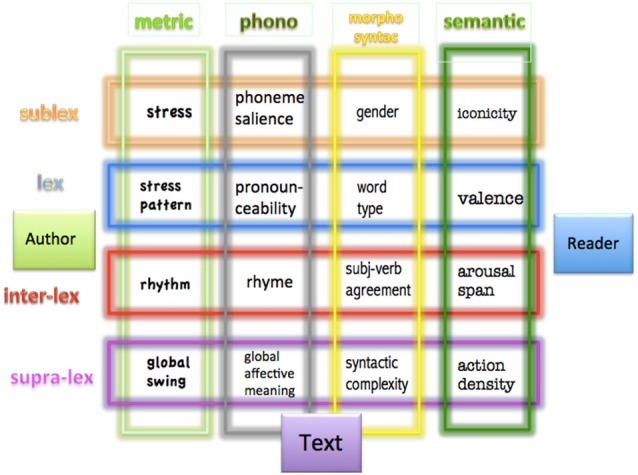
**4 × 4 matrix illustrating four levels of text crossed with four groups of features, with one example feature for each cell of the matrix**.

A good starting point for discussing how to carry out a (textual) structure analysis in (neuro)cognitive poetics research is provided by the works of Jakobson (1960, 1979; cf. Jacobs, 2014 aLoE). In the book “Hölderlin, Klee, Brecht: Zur Wortkunst dreier Gedichte” (Hölderlin, Klee, Brecht: On the word art of three poems), Jakobson (1979) subdivides his observations concerning Hölderlin’s poem “Die Aussicht” (the View), by far the longest of his three analyses, in six sections entitled: time of origin, verse, word types, word repetitions, two expressions of mental derangement, and diotima. Thus, in contrast to his famous quantitative analysis of Baudelaire’s “Les chats” (Jakobson and Lévi-Strauss, 1962), here he also includes a lot of qualitative contextual (historical) factors. Following Jakobson, literary texts can methodically be described by their (1) metric; (2) phonological; (3) syntactic; or (4) semantic properties (and others, of course). Moreover, one can analyze text features hierarchically into (a) sublexical; (b) lexical; (c) inter-lexical (i.e., concerning the relation between two words in a verse, sentence, or paragraph); and (d) supra-lexical (i.e., sentence- and story-level) features (Hsu et al., 2015b; Jacobs, 2015). This forms an initial 4 × 4 matrix, illustrated in Figure 2, that can be extended when required and help guiding research in this field.^3^

At the *sublexical* level, example features that could fill the matrix are: stress (metric), phoneme salience (phonological), gender (morpho-syntactic), or iconicity (semantic). Since phonological features are so important in Jakobson’s work, I will focus on these. Whissell’s (1999, 2000, 2002) pioneering analysis of, phonoemotional profiles’ for several types of material provided empirical evidence for the validity of the assignment of emotional character to phonemes (Jakobson, 1960). Today, there also exists a formal tool for quantifying phoneme salience and basic affective tone (Aryani et al., 2013) which has been used succesfully to show that poems using certain phonological segments more often than expected from everyday language are perceived as more poetic (Ullrich et al., 2015), as well as to predict the general affective meaning (at the supra-lexical level) of poems by the German author Enzensberger (Aryani et al., 2015). This is only one indication that these features can interact at several levels of structure and function and for Jakobson’s (1960) axiom of the interaction between formal (nonsemantic rhetorical) and semantic features, as perhaps best expressed in his famous analysis of the political slogan “I like Ike”: “In poetry, any conspicuous similarity in sound is evaluated in respect to similarity and/or dissimilarity in meaning”. There is also independent evidence that sound structure influences emotional reactions to texts (e.g., Miall, 2001; Wiseman and van Peer, 2003; Auracher et al., 2011), as well as memory performance (Tillmann and Dowling, 2007; Lea et al., 2008).

Considering *lexical* word properties, examples for the 4 × 4 matrix are: stress pattern (metric), pronounceability (phonological), word type (morpho-syntactic), or valence and polysemy (semantic). At this central level of linguistic representation where all other levels converge, one faces the challenge to single out effects of features relevant to neurocognitive poetics, such as emotional valence or subjective beauty, from more than 50 quantifiable factors known to affect (single) word recognition performance (Graf et al., 2005). However, there are tools like the Berlin Affective Word List (BAWL; Võ et al., 2006, 2009) that offer quantitative information on many relevant features of thousands of words, e.g., a dozen relevant psycholinguistic variables, such as word length, neighborhood density, imageability, or frequency, and several affective semantic variables like valence, arousal, or discrete emotion and embodiment ratings (Briesemeister et al., 2011a,b, 2014a,b; Jacobs et al., 2015). Any text can be analyzed “stylometrically” using the BAWL, the, Affective Norms for German Sentiment Terms’ (ANGST; Schmidtke et al., 2014a,b), or similar tools for English (Whissell et al., 1986; Bradley and Lang, 1999; Citron et al., 2012). Examples using the BAWL are the quantification of the *emotion potential* of poems (Aryani et al., 2015), of passages from the Harry Potter novels (Hsu et al., 2015b), or of E.T.A. Hoffmann’s black-romantic story “The Sandman” (Jacobs, 2015; Lehne et al., 2015). Other examples include the stylometric analysis of Beatles songs using the “emotion clock” (Whissell, 1996), of Poe’s poetry (Whissell, 2011), or the prediction of the emotional valence of entire passages or stories on the basis of the valence values of the constituting words (Bestgen, 1994).

At the *inter-lexical* level, example features are rhythm (metric), rhyme (phonological), subject-verb agreement (morpho-syntactic), or arousal span (semantic). Both mean and spread measures of affective lexical variables across a given text passage can be used assuming that they represent different aspects of its *emotion potential* at the level of lexical surface features. The mean of lexical valence and arousal values across a passage may best represent its global emotion potential as a function of the appearance of emotionally consistent concepts. Spread measures, in turn, may better represent dynamic changes or contrasts in readers’ affective experiences and thus meaningfully complement “static” measures like the mean. Mixing words of contrasting valence or arousal in a passage may induce mixed feelings and augment affective responses of surprise, suspense, or curiosity central in structural affect theory of narrative (Brewer and Lichtenstein, 1982). Applying Friston’s (2010) free-energy principle to emotion theory, the affective responses to texts can be seen as the dynamic attribution of emotional valence to every state of the (text) world that an adaptive agent (reader) might visit (Joffily and Coricelli, 2013). Arousal-span (i.e., the range of arousal values of single words across a text segment) and valence-span (the range of respective valence values) appear to be appropriate lexical spread measures serving as proxys for such a dynamic. For example, in the sentence “And then a silence fell over the crowd, from the front first, so that a chill seemed to spread down the corridor” (Rowling, 1999), a high lexical arousal-span is produced by the contrast between the low arousal of “silence” and the high arousal of “chill”, whereas the mean lexical arousal of the whole sentence would be rather moderate (Hsu et al., 2015b). First evidence for the role such inter-lexical variables might play in reading came from data by Lehne et al. (2015) showing that arousal-span can account for about 25% of the variance in suspense ratings from readers of “*The Sandman*” (Jacobs, 2015). Extending this, Hsu et al. (2015b) tested effects of inter-lexical variables (together with lexical and supra-lexical ones) in an fMRI study on reading *Harry Potter* passages showing that they explained additional variance of (supra-lexical) passage arousal ratings. They also found that hemodynamic responses in a number of regions including left IFG, right globus pallidus, thalamus (ventral lateral nucleus and pulvinar), and the left amygdala all showed positive correlations with arousal-span. The robust correlation between arousal-span and emotion-related neural correlates backs the idea that this variable is a promising predictor of emotional experience related to suspense and immersion in reading as hypothesized by the NCPM, providing additional support for the *Panksepp-Jakobson hypothesis*. Recently, Ullrich et al. (2015) found suggestive evidence that minimal valence, another inter-lexical, i.e., relational, feature, can predict the rated sadness of poems.

*Supralexical* features that could fill the matrix are the “global swing” of a language (metric; cf. Schrott and Jacobs, 2011), global affective meaning (phonological), syntactic complexity (morpho-syntactic), and action density or diegetic level (semantic). As far as I know, there is no data-base comparable to the BAWL allowing to quantify relevant supra-lexical variables. As concerns narrative structure, structuralist theorists (e.g., Barthes, Propp) argued that all human narratives have certain universal, deep structural elements in common (e.g., the three-act structure of setup, conflict, and resolution, or, the subject-object, sender-receiver, helper-opponent roles), while poststructuralism claimed that such universal structures were impossible (e.g., Foucault, Derrida). Regardless of which school of thought is correct, researchers in neurocognitive poetics have to be both pragmatic and innovative in their attempts to find suitable descriptors for this level of processing. For instance, they could use tools from quantitative narrative analysis (QNA; e.g., Franzosi, 2010) to operationalize narrative structure and complexity. Examples are the Edmonton Narrative Norms Instrument (Schneider et al., 2006) which allows to compute a textual complexity index based on the number of dependent clauses, or the “Suyzhet” software that can extract plot shapes from novels based on sentiment analysis (Jockers: http://www.matthewjockers.net). Simple measures of supra-lexical syntactic complexity are average sentence length or number of verb phrases. Studying characteristics of sentence length variability using QNA in a large corpus of world-famous literary texts has shown that an appealing and esthetic optimum appears somewhere in between short and long sentences and involves selfsimilar, cascade-like alternation of various lengths (Drozdz et al., 2014). A particularly promising quantitative approach for neurocognitive poetics is Kintsch’s (2012) application of the topic model (Griffiths et al., 2007) allowing to compute the “perfect form” of a text on the basis of measures of complexity (Schmidhuber, 1997), harmony, variety, and dynamic at three levels of description (cf. Solso, 2003): surface level (e.g., form features like rhyme), conceptual level (e.g., topics like “stillness of a morning”), and interpretational level (e.g., metaphorical titles guiding esthetic experiences; cf. Millis, 2001).

In a pragmatic attempt to operationalize narrative complexity of Harry Potter passages, Hsu et al. (2015b) used the number of persons or characters, and the type of inter-character interaction, assuming that the higher this number, the greater the complexity. For example, in the short passage: “Harry waved until the train had turned a corner and Mr and Mrs Weasley were lost from view, the turned to see where the others had got to. He supposed Ron and Hermione were cloistered in the prefect carriage”, there are five characters and one social interaction. However, Hsu et al. did not examine potential effects of this factor, but only used it as a control variable.

Much as for the lexical level, instead of using structural descriptors of texts, rating scales are useful to find out which supra-lexical variables are relevant. Thus, in a reanalysis of the above mentioned data from Jacobs (2015), Lehne et al. (2015) found that the rated amount of action going on in story segments correlated highly with immersion ratings (*r* = 0.95), suggesting that fiction feelings supported by action-rich scenes facilitate immersive processes. In their Harry Potter study, Hsu et al. (2015b) assessed the supra-lexical emotion potential, represented by subjective valence and arousal ratings for whole passages. They reasoned that this variable may go beyond what lexical values alone would predict. As an example, the passage “Ginny glanced round, grinning, winked at Harry, then quickly faced the front again. Harry’s mind wandered a long way from the marquee, back to afternoons spent alone with Ginny in lonely parts of the school grounds.” (Rowling, 2007) was rated as positive, while its mean lexical valence (assessed by the BAWL) was neutral. Here, the supra-lexical emotional impact probably results from the drift of Harry’s mind into the past remembering his relationship with Ginny, that the reader is rather invited to imagine than actually being told about.

Following Dixon et al.’s (1993) proposal of text features, text effects, and the statistical reader, condensed in their statement that “a text is literary if it generates a large number of (common) literary effects in a population”, any of the above discussed “features” or alternative proposals should be submitted to empirical testing and a thorough subsequent evaluation of their relative effect size, before becoming part of a generic catalog to be used in neurocognitive poetics. As evidenced by the results of Hsu et al. (2015b), all these variables can correlate to different extents with both behavioral and neural responses and extensive future research is necessary to disentangle their role in literary reading.

## Elided/Alluded Information and Reader Characteristics

A note on measuring what is absent from texts but still may affect reader responses is in order. In any linguistic discourse, and particularly so in poetic literature, there is a constant interplay of explicitness vs. ellipsis and redundancy vs. ambiguity (Waugh, 1980). Elliptical structures, however, can only exert their intended effects if the signs that have been left out are known to the addressee. In research on single word recognition and reading the influence of (absent) words—partially activated in the hypothetical mental lexicon of readers—on the processing of a printed word has been experimentally demonstrated by Grainger et al. (1989) who discovered the so-called *neighborhood frequency effect*, i.e., the observation that the processing of low-frequency words like BLUR is slowed by the (hypothetical) co-activation in memory of higher-frequency orthographic neighbors like BLUE. The effect was computationally explained (i.e., simulated on a computer) a few years later (Grainger and Jacobs, 1996) and played a significant role in the development of computational models of reading (Jacobs and Grainger, 1994), which in addition to effects of orthographic similarity can also simulate effects of phonological (Jacobs et al., 1998) and semantic features (of absent words) on the processing of a presented word (Hofmann et al., 2011; Hofmann and Jacobs, 2014).

In literature, the well known trope of ellipsis is a straightforward example of a rhetorical feature that works through omission, bypassing the usual mechanism of making meaning from form, i.e., meaning without (printed) form (a mental form representation or anticipation filling-in the elided being likely, though). There are at least nine recognized types of ellipsis (e.g., Kolk, 2001), such as *gapping* (Simon can play the piano, and Marius __the guitar) or *stripping* (Simon can play the piano and Art _ _ _ _, too), and they represent interesting challenges to standard (psycho) linguistic theory, debating, for example, whether ellipsis is a syntactical or semantic phenomenon (e.g., Konietzko and Winkler, 2010). Awaiting a unified theoretical framework for interpreting effects of this trope, neuropoetics researchers can still use structural descriptions (e.g., computing ellipsis frequency per type; Kolk, 2001) or ratings in investigating the role of this interesting text feature, as well as computational models for estimating possible effects of “absent” words on the present ones.

But fiction is abundant of “things not being said” of which ellipsis is only a relatively simple example—and of things left in–or underdetermined, or more or less subtly alluded to. The double indeterminacy (i) between text and reader; and (ii) text and reality characteristic for fiction (Iser), the polyfunctionality of the text in interaction with the polyvalence of the recipient (Schmidt), the polysemantic possibilities and open meaning-gestalts of a literary text (Iser; Holenstein, 1976), the ubiquitious many-to-many correspondence between form and function (or word and object), dubbed the “Proteus Principle” (Sternberg, 2003), the connotative density and wealth of associations (Erlich, 1955), all require that text analyses must be complemented by analyses of variables estimating readers’ “apperceptive mass” (Kintsch, 1980), i.e., their knowledge (e.g., semantic and autobiographical memory), motivations, expectations, preferences, and, generally speaking: personality variables (Jacobs, 2011). Ultimately, neurocognitive poetics research should be able to come up with a theory of the most likely associations and connotations an ideal (or individual) reader (re)produces when reading a given text, i.e., a theory of Proust’s nexus of associations (Epstein, 2004). Analysing the words that make up a text will not do: since “we can only imagine what is absent” (Proust), neurocognitive poetics research needs testable hypotheses about what those things “absent” from a text elicit in a readers’ mindbrain. Barthes’s (1973) insightful analysis of Honoré de Balzacs *Sarrasine* provides a nice example of this phenomenon (cf. Jacobs et al., 2013). A number of words, Barthes compares to “color specks” in pointillistic paintings—seemingly inconsequential pieces of information (e.g., feast, Faubourg, villa), apparently lost in the natural flow of the text—really are meant to evoke a certain (mental) picture in the reader’s mind, to “summon the signified” (in this case the concept of wealth).

Epstein’s (2004) neuroaesthetics theory offers one way of interpreting this phenomenon at the neural level. It is based on Proust’s theory of conscious experience—resembling James’s (1890/1950) division of the stream of thought into a “nucleus” and “fringe” (see also Mangan, 1993)—and his idea that the function of art is to evoke the underlying associative network indirectly in the mind of the observer by using carefully chosen sensory surfaces to control the stream of thought. According to Epstein the Jamesian stream of thought involves distinct neural/cognitive mechanisms, including a network of associations supported largely by the medial temporal lobes (e.g., hippocampus) that determines the relationship between the current nucleus and other potential thoughts and feelings forming the “fringe”. Powerful recent computational models of associative (semantic) memory can help testing the “fringe” part of that theory. For example, Hofmann et al.’s (2011) model can be used to generate hypotheses about how many and which word representations are partially (co)activated in memory when reading a given word. The model succesfully predicted brain activation in left inferior frontal gyrus (LIFG) as a function of computed association strength between words (Hofmann and Jacobs, 2014). With regard to Epstein’s theory, future applications of the model could be to generate hypotheses about which (unconscious) word representations partially activated in the associative nexus forming the “fringe” (like feast, Faubourg, and villa) would finally lead to the conscious evokation of the concept or word WEALTH, as in the above example from Barthes (1973). The model can also be used to simulate the construction of meaning in processing metaphors, for which LIFG has been shown to play a key role (Schmidt and Seger, 2009; Forgács et al., 2012).

In sum, attempts at typologies, taxonomies, computational models, data-bases or other ways to categorize and/or quantify the textual features that theoretically affect reader responses at the various levels outlined above are not sufficient: they need to be complemented by testable hypotheses about how internal processes in the reader’s mindbrain fill-in gaps in the text material through associations that form the basis of memories, imagination, and anticipations, and thus of the three master interests that, according to Sternberg (2003), constitute the universals of narrative: “suspense, curiosity, and surprise, each encoding a distinct functional operation of the mind within narrative’s overall intersequencing, i.e., the dynamics of prospection, retrospection, and recognition, respectively”.

## BG and FG Elements

The simple 4 × 4 matrix lacks a distinction central for dealing with poetic texts: FG vs. BG features. There is an extensive literature on the former from the formalists and structuralists (e.g., Shklovskij, Spitzer, Mukarovsky, Jakobson) to reception-esthetic and linguistic works on poetics and hermeneutics (e.g., Gadamer), and, of course, essays and empirical reports on cognitive poetics (e.g., Martindale, Tsur, Iser, Miall, Kuiken, Cupchik, Oatley, van Peer). In contrast, the (cognitive) poetics literature has paid much less attention to BG features, i.e., the elements of a text that create a feeling of familiarity in the reader (Schrott and Jacobs, 2011; Sanford and Emmott, 2012). However, any literary text always contains a mixture of both BG and FG elements. This can create a tense relation between them and feelings of tension and other affective responses in readers that have been interpreted in terms of the gestalt-psychological principle of figure-ground and may constitute a major future research issue for (neuro)cognitive poetics (Iser, 1976; Van Holt and Groeben, 2005; Schrott and Jacobs, 2011). According to Iser, this tension is created by the fact that the background of a text “includes the repertoire of familiar literary patterns and recurrent literary themes and allusions to familiar social and historical contexts which, however, inevitably conflict with certain textual elements that defamiliarise what the reader thought he recognized, leading to a distrust of the expectations aroused and a reconsideration of seemingly straightforward discrepancies that are unwilling to accomodate themselves to these patterns”.^4^

## BG Features and Immersive Processes

In contrast to FG features, there seems to be no systematic catalog of textual BG features yet. Following Van Peer’s (1986) seminal work, I will therefore take the pragmatic stance considering the issue an empirical one. He described FG as a “pragmatic concept referring to the dynamic interaction between author, (literary) text, and reader”, identifying it through stylistic analysis and operationalizing it at the text level. Thus, each text part that could be identified as prominent due to parallelism or defamilarization (i.e., stylistic devices, rhetorical figures, tropes, or schemes) would count as FG; all other parts would count as the BG against the FG figure. The challenge here is to find the right mix of methods and models allowing to determine with sufficient accuracy and generality which elements induce BG and which FG effects.

In the NCPM, BG features such as familiar words and phrases facilitate immersive processes (absorption, transportation; i.e., the *feeling of getting lost* in a book; Nell, 1988) through the automatic (implicit) activation of familiar cognitive schemata, situation models, and affective responses (e.g., empathy, suspense, or vicarious fear, joy etc.), which correlate with a fluent reading mode (i.e., larger eye movements, shorter fixations) and significant neural activity in the well-known “reading networks” of the left hemisphere/LH (Jacobs, 2011, 2015). Following Iser’s (1976) triadic model, or Ryan’s virtual reality model of immersion in literature (Ryan, 2001), the *immersion potential* of texts depends, among other factors, on *setting* (spatial immersion), *plot* (temporal immersion), and *character emotions* (affective immersion). Thus, texts that offer familiar, easy-to-process spatial aspects, a clear or surprising chain of events providing a good deal of “what happens next?” suspense (cf. Oatley’s, 1994 “Grisham effect”), and, perhaps most importantly, convincing depictions of the inner life of the protagonists (e.g., intentions, emotions, mental conflicts) can drag readers easily into the “text world”, making them forget the “real” environment around them (Jacobs and Schrott, 2015). It is of note that according to a recent survey by Hakemulder (2013), texts are by far the most immersive medium when compared to movies or music. The main reason for this, as reported by Hakemulder’s subjects, lies in empathy-relevant descriptions of the characters’ inner world, followed by plot-related effects of curiosity, surprise, and suspense.

## How can BG Features of Texts and Immersive Reader Responses be Assessed?

**Figure 3:**
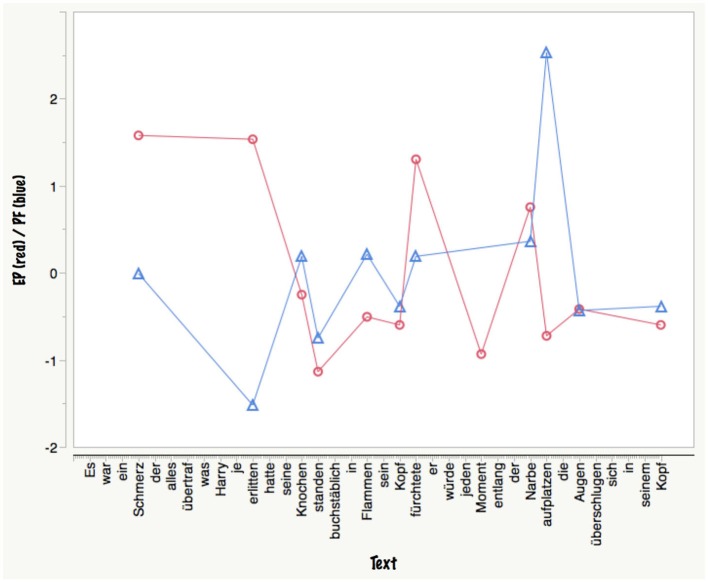
**Example computation of emotion potential (EP = abs(valence x arousal)) and processing fluency (PF = frequency x imageability) for a passage from Harry Potter**. *x*-axis: text; *y*-axis: *z*-values of EP (in red) and PF (in blue). Note thate EP and PF values are only shown for words contained in the BAWL.

Scholars of cognitive poetics (e.g., Martindale, 2007; Toolan, 2008) advocate use of corpus-linguistic methods for stylistic analysis. Martindale’s (2007) “very simple definition of a poetic style” is that “it is a roughly defined lexicon of words that can be used in poetry and a set of rules governing how these words can be used”. Thus, describing the lexical aspects seems a good starting point, like using the Dictionary of Affect to carry out *emotional stylometrics* on various text genres, showing that ratings of excerpts of romantic poetry (e.g., Byron, Keats) on dimensions of pleasantness, activation, romanticism, and preoccupation with nature were consistent with estimations based on the database (Whissell, 2003). However, the lexical aspects alone are insufficient, as discussed above. An example computation of some possibly important BG features (Figure 3) using a short passage in German^5^ from our Harry Potter study (Hsu et al., 2014), illustrates this. Here I computed two lexical composite indices, as provided by the BAWL: *emotion potential*, estimated as the product of valence and arousal values per word (EP = absV × A), and *processing fluency*, estimated as the product of frequency of occurrence and imageability values per word (PF = F × I). Both measures are statistically independent for the chosen passage (*r* = 0). Whereas—all other things being equal—high-frequency, high-imageability words should increase processing fluency and thus possibly contribute to immersive processes, the case of words with a high EP is more complex. On the one hand, they have a high attention capture potential (Hsu et al., 2015b) and thus locally might slow down and hinder immersive processes. On the other hand, depending on inter- and supra-lexical context factors discussed above, globally their summed effect across a passage may contribute to increased fiction feelings, suspense, and thus immersion. Actually, for this passage, the latter was the case since with a mean immersion rating of 4/7 and a mean suspense rating of 4.95/7 it featured among the top 10% and 2.5%, respectively, of all 120 passages analyzed (Hsu et al., 2014).

Thus, higher-level structural and statistical descriptions, for example of the number of familiar concepts, or the ratio of living/inanimate things used in a passage (cf. Jakobson and Lévi-Strauss, 1962), its spatial and temporal aspects (e.g., estimates of action density/number of verbs or action sentences; Hsu et al., 2015b), its event and discourse structure (Propp, 1928/1968; Oatley, 1994) and, of primary importance, depictions of the protagonist’s mental world must complement such data-base guided analyses. While seminal works like Todorov’s (1977), “Poetics of prose”, Iser’s (1976), Act of reading’, or Bortolussi and Dixon’s (2003), Psychonarratology’ (to name only a few) offer interesting perspectives on how to quali- and quantify BG features of texts, I am not aware of any systematic typology or taxonomy that suits itself for purposes of neurocognitive poetics.

One can, of course, tackle the issue empirically by employing scales to determine the BG/FG coefficient of a text, such as adaptations of Van Peer et al.’s (2007) FG questionnaire or the more recent Experiencing Questionnaire (Kuiken et al., 2012). Using their own 12-item Poetry Reception Questionnaire (PRQ) especially designed for analyzing “poetry of mood” (Meyer-Sickendiek, 2011), Lüdtke et al. (2014) recently found evidence for the hypothesis that BG features of German poems from the 18th to 20th century (e.g., Hölderlin, Heym, or Becker), such as the familarity with the depicted motif, phenomenon, or experience (e.g., the stillness of a morning) induced affective reactions and immersive processes in readers, in particular *mood empathy*. A poem by the contemporary German poet Durs Grünbein (Grauzone Morgens; cf. Figure 1 in Lüdtke et al., 2014) constitutes an example for how BG elements like the well known words “Weg” (way) and “Stadt” (city) may activate familiar situation-models in readers facilitating mood empathy, while at the same time line brakes within phrases can be considered deviant FG devices which interrupt reading fluency and create a tension that may result in esthetic feelings associated with closure.

Similar to the related construct of *flow* (Csikszentmihalyi, 1990,^6^; Dietrich, 2004; Weber et al., 2009), *immersion* is far from being a unified concept and this has both theoretical and methodological reasons, i.e., heterogeneity of somewhat entangled definitions, such as *flow, immersion, absorption, transportation, entrancement, narrative engagement, zoning in, or presence*, and the corresponding diversity in operationalizing and measuring the construct. The standard approach is to use scales (e.g., Green and Brock, 2000; Appel et al., 2002; Busselle and Bilandzic, 2009). While these have good psychometric properties, users face the dilemma that they either measure “immersion” *post hoc*, that is *after* the reading act, and thus are prone to memory effects or personal theories about immersion (Weber et al., 2009), or, they try to measure it on-line (either continuously or intermittently), i.e., during the reading act, and then very likely interrupt or stop the immersive experience altogether (cf. Hakemulder, 2013; Jacobs and Schrott, 2015).

Moreover, in general, preverbal or subconscious processes that can accompany elements of immersion, suspense, or defamilarization in reading (Auracher, 1997) must be measured with other methods. Neurocognitive poetics should therefore use complementary, more implicit and objective, methods in addition to the important but, in some respects, limited, explicit verbal report tools. However, “methods must fit the questions” (or phenomena), and in the case of immersion, experimental psychology or cognitive neuroscience so far has little preliminary research to offer which could guide methodological choices. Theoretically, if immersion ratings are highly correlated with suspense ratings (e.g., Jacobs, 2015), peripheral-physiological indicators of states of tension and suspense (e.g., heart rate, EDA, pupillometry) could also be used as indicators of immersive experiences (cf. de Manzano et al., 2010; Keller et al., 2011). The seminal work by Auracher (1997) provided first evidence for this assumption showing clear effects of suspense-related text parts on several peripheral-physiological indicators. The studies by Wallentin et al. (2011) and Lehne et al. (2015) on emotional intensity and suspense effects during listening the “The ugly duckling” and reading the “The Sandman”, respectively, confirm and extend Auracher’s results to the level of brain activity: In Lehne et al., individual ratings of experienced suspense obtained after each text passage were found to be related to activation in the medial frontal cortex, bilateral frontal regions (along the inferior frontal sulcus), lateral premotor cortex, as well as posterior temporal and temporo-parietal areas. The results indicate that the emotional experience of suspense involves brain areas associated with mentalizing (ToM) and predictive inference, aligning with those of Altmann et al. (2014). From the perspective of predictive coding and free-energy theories (Friston, 2010)—which postulate that perception, action, learning, and emotion are based on the minimization of prediction errors, surprise, and uncertainty-suspense can be viewed as the emotional component reflecting this urge for uncertainty reduction.

**Figure 4:**
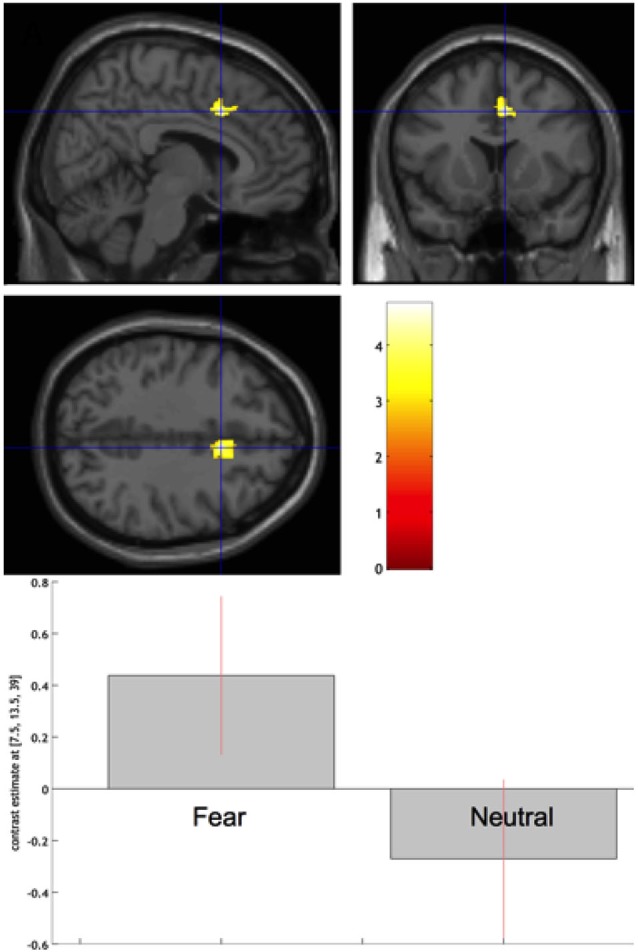
**(Taken from Hsu et al., 2014). Top**: fMRI results: the mid-cingulate gyrus showing a significant correlation difference between passage immersion ratings and BOLD response in the Fear vs. Neutral conditions, cross-hair highlighting the peak voxel (Montreal Neurological Institute coordinate [*x*, *y*, *z*] = [8, 14, 39]). The color bar indicates *t*-values. **Bottom**: The estimated response strength in the peak [8, 14, 39] for both experimental conditions. The error bars represent 90% confidence intervals.

However, neither Auracher, nor Wallentin et al., or Lehne et al. measured *immersion*. First evidence that similar brain regions are involved in immersive experiences and feelings of suspense comes from a study by Hsu et al. (2014) on immersion in passages from Harry Potter novels. In line with the *fiction feeling hypothesis*, immersion ratings were significantly higher for fear-inducing than for neutral passages, and hemodynamic activity in the mid-cingulate cortex correlated more strongly with immersion ratings of fear-inducing than of neutral passages. Thus, descriptions of protagonists’ pain or personal distress featured in the fear-inducing passages might have caused increasing involvement of the core structure of pain and affective empathy the more readers immersed in the text (see Figure 4). Although this is rather speculative, the predominant locus of effects in the mid-cingulate cortex allows the assumption that immersive experiences are particularly facilitated by the motor component of affective empathy, at least for the Harry Potter or similar materials, which feature particularly vivid descriptions of the behavioral (expressive) aspects of emotion. So far, Hsu et al.’s (2014) study is the only one looking for neural correlates of immersion in reading (but see Wilson-Mendenhall et al., 2013, for social immersion) and further research—combining verbal, peripheral-physiological, and neuroimaging methods in comparative studies (Schlochtermeier et al., 2015)-is needed to shed more light on this fascinating process. In this respect, Dietrich’s (2004) *hypofrontality hypothesis* of flow-states, or Weber et al.’s (2009; Klasen et al., 2012) alternative theory of cognitive synchronization of attentional and reward networks might be interesting views to follow.

## Assessing FG Features and Esthetic Processes (Jakobson’s Poetic Function)

In the NCPM, FG features are assumed to facilitate esthetic processes through attention capture, adaptation of schemata and situation models, construction of new meaning gestalts, self-reflection, or concernedness. These are assumed to correlate with a dysfluent reading mode (i.e., smaller eye movements, longer fixations), and significant neural activity in right hemispheric networks and the ancient play and lust circuits (Jacobs, 2011, 2015).

On the text side, lexica of rhetorics, such as Lausberg’s (1990) handbook, may offer useful theoretical classifications and typologies for potential FG features, whose value for empirical sciences, however, might be limited (McQuarrie and Mick, 1996). Still, considerable efforts to formally determine what is perhaps the best studied figure of speech, i.e., metaphors, seem to produce first results, as evidenced by the Pragglejaz group’s method for detecting metaphorically used words in discourse (Pragglejaz Group, 2007), or Kintsch’s computational model of metaphor comprehension (Kintsch, 2000). A valuable tool, the database by Cardillo et al. (2010), is explicitly designed for testing neural hypotheses about metaphor processing, offering quantitative estimates of over a dozen variables such as familiarity, imageability, or figurativeness. Similar databases exist for proverbs (Bohrn et al., 2012a), or idioms (Citron et al., 2015). Recently, Jacobs et al. (2013) proposed an “abstractness scale” especially designed for structural descriptions of poems. Based on literary theory (Meyer-Sickendiek, 2011), the tool offers nine scales most relevant for interpreting lyrical texts and judging the degree of abstractness or defamiliarization (“Verfremdung”). Table 1 shows example ratings of expert judges for two German poems, Eduard Mörike’s “An einem Wintermorgen” (On a winter morning, 1825) and August Stramm’s “Der Morgen” (The Morning, 1914). In principle, the scale (or adaptations of it) can also be applied to other text parts, for instance, to test the NCPM’s prediction that higher degrees of FG should correspond with higher ratings of esthetic emotions/beauty. While the results of a pilot study reported in Jacobs et al. (2013) did not support this simple prediction for a corpus of 24 poems, the elaborated statistical analyses by Lüdtke et al. (2014) using their PRQ on a better controlled set of 12 poems showed that esthetic liking was best predicted from two compound FG features (style and form).

**Table 1:** **Degree of abstraction for two poems analyzed in Lüdtke et al. (2014)**.

Scale with verbal anchors (1 = classical, 5 = abstract)	Eduard Mörike “An einem Wintermorgen”	August Stramm, “Der Morgen”
Meter (regular—without)	2	5
Rhyme (regular—without)	2	5
Rhythm (flowing—dammed)	1	5
Representativeness (illustrating—alienating)	1	5
Motive evaluation (inflating—degrading)	1	5
Language/Grammar (conform—deviating)	2	5
Mimesis (ingressive—abstract)	2	5
Subjectivity (explicit—transsubjective)	1	5
Montage (scant intertextual references—strong intertextuality)	3	4
Mean abstractness/defamiliarization	1.67	4.56

Another, more general, potentially useful tool is the relatively simple taxonomy of rhetorical figures proposed by McQuarrie and Mick (1996). It offers parsimonious hypotheses about possible effects of schemes (e.g., rhyme, anaphora) and tropes (e.g., hyperbole, metaphor) on reader responses (e.g., increased attention, liking), ordering them along two gradients (complexity and artful deviation). For example, if measures of attention capture or liking were a linear function of the gradient of artful deviation (from some standard), then this framework would predict the following rank order of effect sizes: metaphor > hyperbole > antimetabole > rhyme. Even though in the light of work like Berlyne’s (1971) such a simple linear relationship seems highly unlikely, it could serve as a null-hypothesis for neurocognitive poetics studies against which more sophisticated assumptions could prove their validity.

With regard to esthetic liking, a recent neurocognitive study by Bohrn et al. (2012a, 2013) suggests that things might be more complicated indeed. It looked at the neuronal correlates of esthetic liking of original and artfully deviated (i.e., defamiliarized) German proverbs like “All roads lead to Rome” or “All sins lead to Rome”, respectfully, featuring numerous proverb-characteristic rhetorical elements: phonological similarities (rhyme/alliteration), meter, parallelism, brevitas (artful shortness), or ellipses. A first result was a positive correlation between explicit beauty jugdments and stimulus familiarity supporting the *preference for familiarity effect* and the hypothesis that processing fluency due to familiarity contributes to beauty judgments (Reber et al., 2004; Kuchinke et al., 2009). However, the correlation accounted for only about 30% of variance in the beauty judgments leaving 2/3 of variance to be explained by other factors. What is more, the observed correlation might also be explained with regard to the historical evolution of the material: A familiar proverb may be familiar precisely because specific esthetic qualities account for its cultural success.

**Figure 5:**
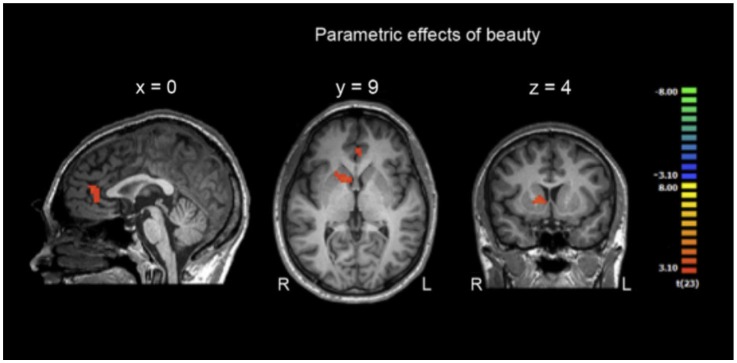
**(Taken from Bohrn et al., 2013)**. fMRI results showing parametric effects of beauty evaluation in the right caudate nucleus extending to putamen and anterior rostral part of the MFC. (voxel height threshold at *p* < 0.005, cluster width threshold of 24 voxel).

The main result of the study supported the *Panksep-Jakobson hypothesis* and the NCPM: the parametric hemodynamic responses of two brain regions associated with reward and beauty (Vartanian and Goel, 2004) shown in Figure 5 below, the caudate nucleus of the ventral striatum and a part of the ACC, indicate processes of spontaneous esthetic evaluation during sentence reading.^7^ Thus, the ancient neural circuits associated with reward and pleasure, as described in Panksepp’s (1998) emotion theory, also seem at work when humans perform one of the most complex and unnatural skills the mind-brain is capable of: reading (Kringelbach et al., 2008). Moreover, they might be directly linked to the “poetic function” of Jakobson’s (1960) extended version of Bühler’s (1934) organon model of language functions (see Figure 1 above). The rewarding character of novelty and FG through artful deviation has long been recognized also in literary theory, for example in Barthes’ statement about the “pleasure of the text” the reward that comes from processing a clever or novel arrangement of signs, or in Iser’s “pleasure” in closing an open meaning-gestalt (cf. Jacobs, 2011). According to Berlyne (1971) incongruity or deviation can produce a pleasurable degree of arousal, one of two variables that determine affective reactions.

The foregrounded proverb variants present a nice example of how BG and FG features can be combined in a single sentence, why it seems appropriate to analyze potential FG effects with regard to the BG features contained in the stimuli, and why the FG construct should be treated as a complex, continuous multidimensional variable: Due to their multiple rhetoric features, all proverbs can be considered FG elements of language if seen against a BG of non-rhetorical, non-figurative control sentences. However, while the memory of the original proverb (“All roads lead to Rome”) provides familiar BG information the one-word change (“sins”) of the altered variants was intended to create a FG effect. The tension thus created and the resulting affective and esthetic reactions should vary with the degree of familiarity of the proverb and the degree of novelty, deviation, incongruity, or originality of the altered word or version; a hypothesis to be tested by future work. A further complication apart from determining the optimal BG/FG ratio of verbal stimuli lies in the fact that almost all verbal stimuli possess several features that, given the appropriate context, may count as FG, raising questions about the additive vs. interactive nature of FG effects or the dominance of features at different levels of structure and processing, e.g., phonological vs. semantic.

Even single words can already make the task of determining which potential FG features contribute to esthetic effects rather complicated. Intuitive evidence for this is provided in the book on the most beautiful German words (Limbach, 2004), in which, for example, the nine year old Sylwan Wiese explains why the German word LIBELLE (dragonfly) is the most beautiful for him: “it has three “Ls” which is his preferred letter. This makes the word glide so well on his tongue (which is not the case for all German words). He also loves seeing them wobble and finds that the word expresses this feeling, that it ensures that one is not afraid of these insects”. A deeper analysis uncovers more cues like the fact that the first four letters (LIBE-) phonologically form and perhaps unsconsciously evoke the German word for “love” (LIEBE), or that the last four (-ELLE) conjure feminine associations. Importantly, the child already mentions three cues for the beauty of words, an articulatory-phonological one (the gliding Ls), a sensorimotor-perceptual one (the wobbling), and an affective-semantic (no fear). This supports the view that both associations with discrete emotions and embodied cognitions play a role in esthetic appreciations of words (Louwerse, 2011; see also Jacobs et al., 2015, for a recent empirical demonstration using the BAWL). According to Louwerse’s (2011) symbol interdependency hypothesis language comprehension is both symbolic and embodied: it can be symbolic by bootstrapping meaning through relations between the symbols, but it can also be embodied through the dependencies of symbols on indices and icons. On the other hand, similar to the proverb case, the child’s description also raises the question whether one of the three mentioned cues is more important than the others for the “poetic function” or whether they add up (or interact) in forming the feeling of beauty, according to Fechner’s (1876) concepts of threshold level and interaction.

A recent behavioral study using the same database of over 800 German proverbs as in the Bohrn et al. studies looked into this by investigating effects of three rhetorical variables and potential FG features, rhyme, meter, and brevitas (i.e., artful shortness), on ease of comprehension and beauty ratings, as well as persuasiveness choices (Menninghaus et al., 2014). A main result was that while completely de-rhetorized versions of the sentences (i.e., where brevitas/meter and rhyme were deactivated) were judged to be easiest to understand, the fully rhetorical versions having all three features were judged as most beautiful, the presence of the FG feature rhyme being apparently more important than meter and brevitas. The observed interactive effect of the three features on beauty ratings seems to call for a revised version of Fechner’s and Jakobson’s notions introducing a hierarchy of FG features or a more differentiated, process-oriented variant of the “poetic function” of language.

Much as for BG features and immersion, the standard method of measuring FG features and esthetic experiences, judgments, or emotions in empirical esthetics is the use of (explicit) tools such as Likert scales for measuring emotional valence, liking, or beauty (e.g., Leder et al., 2004), or as concerns literature reception, such as Hoffstaedter’s (1987), Martindale and Dailey’s (1995), or Van Peer et al.’s (2007) scales (see Sopcák, 2007). Implicit measures using oculo- and pupillometric or brain-electrical methods are also increasingly used (e.g., Pynte et al., 1996; Kuchinke et al., 2005, 2009; Võ et al., 2008; Augustin et al., 2011; Scheepers et al., 2013). Words of lower frequency or predictability attract longer fixations and more regressions (Rayner, 1998) and since foregrounded words usually fulfill these criteria, eye tracking is a promising method for neurocognitive poetics. Another is EEG: following Kutas’ seminal work on N400 amplitude modulations by semantic anomalies in sentences (see Kutas and Federmeier, 2011, for review), Pynte et al. (1996) were among the first to demonstrate the potential of this ERP component for studying the time-course of the “poetic function” of language using metaphors, albeit rather non-poetic ones, as material. In the same year, the German poet Durs Grünbein (1996) introduced the term “factor N400” as a general proxy for FG features as “brainphysiological attention catchers”, and an index of the FG potential of metaphors, such as “urne and uterus” or “term of endearment and cruelty”. He formulated that such metaphors cause “neurolinguistic clashes” and called for a poetry full of images rich in “factor N400”. His poem “*O Heimat, zynischer Euphon”* (O homeland, zynical euphon) is supposed to be a case in point (Ganseuer, 2006). Indeed, all other things being equal, the amplitude of the N400 decreases with increasing predictability of a word in a sentence, idiom, or story (e.g., Van Berkum et al., 2005; Dambacher et al., 2006, 2009, 2012; Vespignani et al., 2009), an effect that recently was replicated using fNIRS (Hofmann et al., 2014). Both eye tracking and EEG methods can be combined to provide a more ecologically valid means of studying literature reception, but the methodology is still not fully developped (Hutzler et al., 2007; Dimigen et al., 2011).

Finally, researchers interested in neuroaesthetics and neurohumanities also use neuroimaging studies (e.g., Jacobsen et al., 2006; Cupchik et al., 2009; Chatterjee and Vartanian, 2014), the latter consistenly showing that the pleasure that people experience from looking at beautiful objects (including sentences; Bohrn et al., 2013) automatically recruits parts of the brain’s general reward circuitry (Kühn and Gallinat, 2012), thus confirming the *Panksepp-Jakobson hypothesis*. In their meta-analysis, Bohrn et al. (2012a) also found a significant activation of the left amygdala for (foregrounded) figurative as compared to literal language which can be taken as a sign for higher emotional relevance (Sander et al., 2003) and/or affective intensity (Phan et al., 2004) of the former. The above mentioned LIFG turned out to be the structure with the largest effect distinguishing between figurative and literal language processing, its contribution being significantly larger for metaphor and idiom than for irony/sarcasm processing.

In what is perhaps the first published fMRI study on (printed) poetry reception, Zeman et al. (2013) found that brain activation increased with increasing literariness (ratings which they viewed as a measure of FG features) in predominantly left-sided regions, including the precentral gyrus, and areas of the basal ganglia. They interpreted these basal ganglia activations as likely reflecting the increased processing demands imposed by linguistically demanding texts, the basal ganglia having been shown to also play cognitive roles. Their activation in the LH by “literariness” was seen in line with evidence that these structures are engaged by complex syntax and semantic ambiguity. Experimenter-chosen (self-selected) poetry activated brain regions that have been associated with introspection, autobiographical memory, prospection, ToM, and the default mode: the right cingulate gyrus, left superior temporal gyrus, both hippocampi, and the right temporal pole. The latter activation by poetry was linked to “coherence building” or “propositionalization”, the process by which coherent meaning is constructed on the basis of prosodic, syntactic, and lexical cues (Ferstl et al., 2008).

### Context and Reader Aspects

Understanding reading as motivated, goal-directed behavior, it is clear that numerous intentions of many different qualities (e.g., information seeking, curiosity, decision help, reviewing, typographical error finding, pleasure, mood management, etc.) can determine which piece of text is chosen (e.g., genre decision) and how it is processed (e.g., slow letter-by-letter scrutinizing vs. quick scan or deep, reflective reading). Literary genres and text types (e.g., fairy tales, crime stories, poetry, etc.) act on what Miall and Kuiken (1998) have termed the “formalist contract, according to which “A reader taking up a literary text thus makes several related commitments that guide the act of reading.” Indeed, both the choice of the reading medium (e.g., printed vs. digital books) and genre have been shown to affect the reading process at all three levels of inquiry (i.e., the neuronal, subjective-experiential, and objective-behavioral; see Jacobs, 2011, for a summary). Moreover, reading provides learning opportunities for simulating the social world and thus promotes the interpretation of social information and progress in emotional skills (Mar and Oatley, 2008). In her enlightening paper “One Lesson Learned: Frame Language Processing—Literal and Figurative— as a Human Brain Function”, Kutas (2006) argued that when language is properly appreciated as one brain function among many, psycholinguistics will benefit from heeding certain factors that have received proportionately little attention within mainstream psycholinguistic research: the hemispheres, time and timing, context liberally construed to include, for example, personality traits and mood, and individual differences as a proxy for experience. The same holds, in an even stronger way, for neurocogitive poetics.

Two examples of neurocognitive experiments show that both the reading mode and reader personality factors influence reading-related behavioral *and* brain activity. Altmann et al. (2012, 2014) tested the hypothesis that the same text would be processed differently depending on whether participants believe it to be *factual* or* fictional*. Using short narratives with highly negative content and neutral controls, they showed that reading in a factual mode engaged an activation pattern suggesting a past-oriented, action-based reconstruction of the events depicted in a story. In contrast, brain activation patterns corresponding to reading fiction seemed to reflect a constructive simulation (imagination) of what might have happened. Another study by Nijhof and Willems (2015) supports the idea of the existence of qualitatively different styles of moving into literary worlds. Their study provided on-line neural evidence qualifying how people differ in their engagement with fiction by showing that some people are mostly drawn into a story by mentalizing about the thoughts and beliefs of others, whereas others engage in literature by simulating more concrete events such as actions.

To summarize, in the model discussed below I assume that competent readers use their experience, knowledge, and motivation to make genre-specific text choices and accordingly take a reading perspective which co-determines their reading mode/behavior (cf. Mar et al., 2011).

### Cognitive, Affective, and Aesthetic Theories and Models for Neurocognitive Poetics

The role of structural and process models for research in (cognitive) poetics and the necessity of inter-disciplinary collaboration was already highlighted by Van Dijk (1979, p. 605), e.g., “This means that the literary theorist should collaborate with the psychologist in order to develop processing models involving both a detailed structural analysis of these discourse types and the specific demands of the context and their consequent processes of comprehension and representation in memory”. Together with the cognitive psychologist Walter Kintsch, van Dijk pioneered this approach with models of text comprehension and production (e.g., Kintsch and van Dijk, 1978). In the following I will provide a brief sketch of relevant literature which should be of potential use for neurocognitive poetics and which definitely influenced the NCPM discussed afterwards.

#### Cognitive Theory

Following Kintsch and van Dijk’s work, a wealth of cognitive models and theories relevant to (neuro)cognitive poetics enriched the literature on reading and related fields like disourse or metaphor processing. Here I can only mention a selection (see Jacobs, 2011, for an extensive list of literature covering the fields of cognition, emotion, (psycho)-linguistics, and poetics). Among the early works, Bower’s (1981) work on mood, Just and Carpenter’s (1980) reading model, Graesser’s (1981) or Groeben’s (1982) books on prose comprehension and reading psychology, Johnson-Laird’s (1983) work on mental models, or Ortony et al.’s (1978) piece on metaphor processing are prominent examples, the former representing a notable exception to the mainstream work that focused on “cold cognition” remaining silent with regard to affective or esthetic processes. Gernsbacher’s (1990), Zwaan’s (1993), Gibbs’s (1994) or Gerrig’s (1998) books also continue to be influential. All these represent general theories allowing to account for behavioral data in a rather qualitative fashion. Such work can be usefully complemented by more specific computational process models which allow to quantitatively predict behavioral data, among which extant models of word recognition (e.g., McClelland and Rumelhart, 1981; Grainger and Jacobs, 1996), eye movement control in reading (e.g., Engbert et al., 2005), or text processing (Kintsch, 1988, 2000). It is of note that although the Kintsch and van Dijk (1978) model aspired to be a general theory of text comprehension and production, Kintsch (1980) recognized quite early that it is restricted in its scope and applicability in many ways (especially as concerns (neuro)cognitive poetics), and later extended the model to include such aspects relevant to literary text comprehension, e.g., metaphor processing, and beauty (Kintsch, 1994, 2000, 2012).

#### Emotion Theory

Bridging the language-emotion gap being a central goal of neurocognitive poetics, emotion theories connectable to reading are important. While most theories of emotion remain as silent with regard to language processes, as most (psycho)linguistic theories keep still about emotions (for an exception see Schwarz-Friesel, 2007), two seemingly opposed theories have useful potential: Panksepp’s (1998) neuroaffective theory which has been related to language (Panksepp, 2008), word recognition (Briesemeister et al., 2014a,b), and reading (Jacobs, 2011, 2015), and Feldman Barrett’s constructivist theory (e.g., Barrett et al., 2007) or Koelsch et al.’s (2015) “Quartet” theory which explicitly refers to language processes (albeit not reading). Earlier relevant work includes Oatley and Johnson-Laird’s (1987), or Ortony et al.’s (1988) theories of emotion.

#### Aesthetic Theory

Empirical esthetics started with Fechner (1876), and after some early theoretical works, such as Birkhoff’s (1932), and Eysenck’s (1941), was boosted by Berlyne’s (1971) ground-breaking “Aesthetics and psychobiology”. Among recent work, the most influential with regard to the NCPM discussed in the next section, is Leder et al.’s (2004) seminal process model of esthetic appreciation and judgment. Other prominent work comes from Kreitler and Kreitler (1972), Goodman (1976), Martindale (1988), Cupchik and Laszlo (1992), Solso (1994, 2003), Maffei and Fiorentini (1995), Ramachandran and Hirstein (1999), Tyler (1999), Zeki (1999), Chatterjee (2004), Epstein (2004), Jacobsen et al. (2004), Jacobsen (2006), Fitch et al. (2009), or Kintsch (2012).

### The NCPM

Apart from the theories mentioned above many other works from classical rhetoric to modern linguistics are relevant for the present topic and have influenced the NCPM, but for reasons of journal space I must refer to our book on brain and poetry for a comprehensive treatment of this work (Schrott and Jacobs, 2011). The NCPM, developped originally on over 30 pages in the last chapter of this book, represents an eclectic, comprehensive effort to synthesize ideas and results from various disciplines including rhetoric, esthetics, and poetics, experimental reading research, or cognitive and affective neuroscience in an attempt to bridge the language-emotion gap and to go beyond “cold” information processing models by including “hot” affective and esthetic processes into a general framework. The model belongs to what I have called the verbal or V-type (“boxological”) category elsewhere, i.e., it is a prequantitative, descriptive model with all the corresponding pros and cons (see Jacobs and Grainger, 1994; Jacobs and Hofmann, 2013; Hofmann and Jacobs, 2014; for a discussion of these) The main reason for this lies in the fact that as far as I can tell the data base in neurocognitive poetics research still is too sparse to motivate more formal model types, e.g., algorithmic or mathematical models.

Thus much like Leder et al.’s (2004) and Chatterjee’s (2004) models of (neuro)esthetic processing, or Koelsch and Siebel’s (2005) neurocognitive model of emotions in music, the NCPM represents a first step towards more specific formal modeling by making explicit—and thus testable—a number of hypotheses about mental processes theoretically involved in (written) literature reception and their interrelations at the three main levels of inquiry (i.e., the neuronal, subjective-experiential, and objective-behavioral ones). A full treatment of the NCPM being beyond the scope of this paper (see Jacobs, 2011, 2014, 2015; Jacobs et al., 2013), after a brief sketch I shall concentrate on discussing recent and future developments, limitations and challenges.

**Figure 6:**
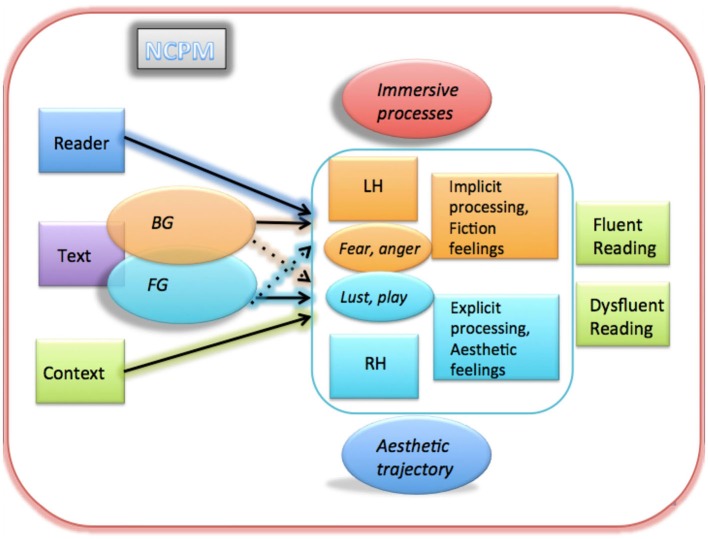
**Simplified sketch of the NCPM (adapted from Jacobs, 2011, 2014, 2015) with a fast upper route triggered by BG text elements, and a slower lower route responding to FG elements (see text for explanation)**.

The highly simplified sketch of the model presented in Figure 6 starts on the left side with the three general factors determining readers’ mental and behavioral responses to literary texts: text, context, and reader variables. Above, I have given examples for how all three factors can affect the reading act at the three processing levels. Concerning text, the model’s central hypothesis is that BG and FG features activate (at least partially) distinct neural networks and cognitive-affective processes (i.e., the upper and lower routes) with measurable behavioral and neural effects. The former would usually activate the fluent, mainly LH-controlled reading mode and immersive processes, supported by the ancient affective core systems described by Panksepp (1998, 2008), in particular fear and rage (anger) that may be involved in fiction feelings and the built-up of suspense. In contrast, FG features usually would induce slowed reading and facilitate esthetic emotions through increased RH contributions, supported by the ancient lust and play systems. However, this basic distinction is not a black and white one, but a matter of degree. Already in the rather speculative, underdetermined original model^8^ I had pointed out that the behavior of complex nonlinear dynamic systems like brains, cannot adequately be described by such mutually exclusive categories, but that it is difficult to graphically represent dynamic processes that can overlap both in (neuroanatomical) space and time otherwise than by “boxological” models (Jacobs, 2011). Thus, as much as BG and FG features can overlap within a text (or even single words and phrases, as shown above), the processes triggered by such text elements can also overlap. For example, the affective “play” system of the lower FG route of the model, assumed to facilitate esthetic feelings, may also take part in pleasurable immersive processes (i.e., ludic reading, Nell, 1988). Nevertheless, the probabilistic predictions of the model are testable and have been examined in several behavioral and neurocognitive studies, discussed above. Also, although the strongest falsificator of the model, i.e., proving that generally subjects immerse in texts full of FG features or, in contrast, experience esthetic feelings when reading texts completely devoid of FG features, cannot be tested categorically, the model would still be “falsified”,^9^ if a majority of data showed this to be correct on average.

So far, the evidence of several studies testing core assumptions of the model generally supports the basic BG/FG distinction, as well as the fiction-feeling and Panksepp-Jakobson hypotheses, but it is still too early to draw any conclusions about its general validity (e.g., the studies by Altmann et al., 2012, 2014; Bohrn et al., 2012a,b, 2013; Forgács et al., 2012; Hsu et al., 2014, 2015a,b,c; Lüdtke et al., 2014; Aryani et al., 2015; Jacobs, 2015; Lehne et al., 2015). We have some evidence that answering the question of whether the NCPM’s prediction that higher degrees of abstractness or FG correspond with higher ratings of esthetic emotions/beauty is correct, depends much on stimulus, task, and context conditions and, of course, the way FG and esthetic emotions are operationalized (see above; Jacobs et al., 2013). As concerns the laterality assumption, the results of several studies also draw a mixed picture. While some language functions clearly are lateralized (Kutas, 2006), I would consider the issue of a more pronounced contribution of the RH to the processing of figurative language (e.g., graded-salience hypothesis by Giora (1997); coarse semantic coding hypothesis by Jung-Beeman (2005) to remain an open one, given that four relevant meta-analyses are unconclusive, suggesting that the RH only shows significant effects in metaphor processing when metaphorical meaning is novel, when it is presented in sentential context, when the task is semantic relatedness judgment (Rapp et al., 2012; Vartanian, 2012; Bohrn et al., 2013; Yang, 2014; see also Ferstl, 2010; Schmidt et al., 2010). Thus, conventionality, contextual complexity, and task demand matter, and a number of other factors may be responsible for this mixed bag of results (Lai et al., 2015). For example, Forgács et al. (2012) found evidence that previous studies might have found RH activations mainly because of semantic distance processing, but not because of metaphoricity. Thus, the last word on the laterality assumption built into the model is not yet spoken.

### Literary Reading According to the NCPM

In a nutshell, the NCPM describes literary reading as follows (cf. Jacobs, 2011): already during the selection of the reading material psychological context factors such as mood and motivation play a role. If a reader engages into a formalist contract with author and text (Miall and Kuiken, 1998), this will cause a genre-specific literary reading perspective characterized by a generally slower reading tempo, due to increased interest in and attention to surface or formal FG features: this reader is ready to fulfill Jakobson’s “poetic function” becoming open and sensitive to literary figure-ground constellations. However, this is no all-or-none decision. The contract with a poem by, say, Celan must and cannot be the same as the one with a novel by Tolstoy. Perhaps a pure or dominant “poetic reading mode” can be maintained throughout an entire poem, but hardly during an entire novel. In the extreme, perhaps our brains cannot even maintain it during reading a single line, such as Celan’s “Schwarze Milch der Frühe, wir trinken sie abends” (“Black milk of daybreak we drink it at evening”). For the German word “der” in this oxmoron, as much as the other three words, cannot “not be read”, unless one closes the eyes. Once reading has been learnt, one is “doomed” to do it automatically. Not every word is read in an equally fluent way (some are skipped, too): Familiarity, predictability, pronounceability, word type, case role, number, frequency and opacity of morphemes, imageability, associative density, semantic cohesion, and a wealth of other quantifiable factors determine single word recognition and thus the general reading tempo. Moreover, what Bühler (1934) called the “spheric fragrance” of a word, will play a role: words and the corresponding (embodied) thoughts and feelings are “substance-controlled” (Jacobs et al., 2015), meaning that each word likely activates different functional brain networks, perhaps even never exactly the same twice, and surely “schwarze Milch” will generate a different neural (and oculomotor) activity from “der”. The (re)constructive mental processes are different, and it is likely that “der” will not be fixated by the wandering gaze at all, its meaning being inferred from context, while the eyes, after having fixated the word “Frühe”, might well jump back to the word “Schwarze”, to provide more sensory information for the brain to close the “meaning gestalt” offered by Celan’s famous oxymoron. Whether a reader (esthetically) appreciates this rhetoric figure as a felicitous image, idle babble, or a disgust-arousing oxymoron depends on many more factors than the model can currently specify.^10^ To what extent a reader is concerned by these words, finds them beautiful or ugly, becomes interested in reading the rest of the poem (and possibly more of Celan) after this first line, or engages into a self-altering reading act (Kuiken et al., 2004, 2012), depends as much on his apperceptive mass (Kintsch, 1980) and many other factors as on the “poeticity” or BG/FG coefficient of the line.

Still, if a given line or text segment contains primarily BG elements, i.e., familiar words, images, socio-cultural codes, situation models or affective scripts, and thus is interpretionally shallow, i.e., offering a high semantic transparency and interpretation potency (Dixon et al., 1993)—as in some of the above examples from Harry Potter-, then the reading act will be dominantly controlled by the well described LH reading system (Jacobs, 2011). This does not mean that the RH is switched off, but that its language-related functions are relatively more silent than when processing text full of FG elements. In reading BG dominant texts, the two fundamental processes underlying reading, word recognition and eye movement control, are little disturbed by attention-capturing features and the higher cognitive processes supporting reading, like mental situation-model and event-structure building (Kintsch and van Dijk, 1978; Zwaan, 1993; Speer et al., 2007) go on automatically without much effort. On the affective side, the BG-related feeling of familiarity creates the basis for typical scenarios of fiction feelings: empathy for characters or events, sympathy for a protagonist, suspense and occasionally increasing curiosity and arousal in the context of the “what happens next” question, or hope concerning a positive outcome and joy/relief when it has happened. Hypothetically, the fear, rage/anger, and also the care and panic systems from Panksepp’s (1998) theory play a bigger role here than lust, play, and seek, associated with the model’s lower route. If a critical number of such factors work together, the feeling of immersion can result: the reader is absorbed by and transported into the text world, being in a “flow”, and “in the middle of the text” (Iser, 1976).

In contrast, when more and more unusual form elements and semantic ambiguities pop-out of the text passage, the standard affective and cognitive schemata only rarely suffice to “make meaning” (i.e., the ultimate goal of reading), and mixed feelings, esthetic emotions, and (self-)reflective thoughts oust the general feeling of familiarity, engaging the lower route. Presumably, then the activity in the left dorsolateral reading circuit (e.g., LIFG) has increased as has the action of the lust, play, and seek systems, and of the RH’s associative networks. The reader is now in a “poetic mode” of tacit evaluative processing, reading the text parts in a FG manner, i.e., not only (automatically) recognizing words, but “seeing”, “hearing”, or “smelling” them. Eye movement behavior slows down, as do thoughts and feelings: they expand and possibly later also do self-perception and personality. The process of closing “meaning gestalts” requires this, because the multitude of “meaning potentials”, the author has subtly created, allows to discover or construct various new ones (Iser, 1976). The reader encounters her/himself, is concerned, because “the familiar” is “outperformed” (Gadamer, 1964/1985). But the reward for so much effort already lingers at the end of the “esthetic trajectory” (Fitch et al., 2009): after initial moments of familiar recognition, followed by surprise, ambiguity, and tension, the closure of meaning gestalts and tension, full of relish, results from processes of integration and synthesis, occasionally supplemented by an “aha” experience (Qiu et al., 2008) or *feeling of good fit*, “rightness”, or harmony which accompanies an esthetic feeling motivating to continue to read and thus closing the reading circle of the model (Mangan, 1993, 2008; Lavazza, 2008; Jacobs, 2011; Kintsch, 2012).

In conclusion, the current state of the art in neurocognitive experiments on literary text reception does not allow any firm decisions with regard to the validity of the dual-route model’s main assumptions, while others must remain rather speculative, due to a big lack of empirical data or methodological limitations (e.g., registration of ocular, brain electrical or hemodynamic activity during natural, hour-long reading). But, whether verified or falsified, if the still very inclusive model continues to contribute to a sharpening of conceptual and methodological tools and the generation of more specific hypotheses and future research, it would have fulfilled its function (Jacobs and Grainger, 1994).

### Some Challenges

In my above discussion of methods and models for neurocognitive poetics, I have pointed out several challenges, worthwhile to be recapped. On the theoretical side, going beyond mainstream “cold” information processing models, neurocognitive poetics needs to integrate concepts from the humanities with current models from cognitive psychology and linguistics, affective neuroscience, and emotion theory, to develop “hot” process models that offer ecologically more valid and more specific hypotheses than the standard models. Inspired by van Dijk’s and Kintsch’s early transdisciplinary program, the outstanding works by Bühler and Jakobson, and the more recent ground-breaking research by van Peer and others (see above), the NCPM framework I have developped over the last 5 years only provides a first hint into—hopefully—the right direction.

The apparent limitations of the NCPM represent a basis for defining future challenges. A first one is developping testable hypotheses on how specific reader and context variables interact with text factors. For example, readers unaware of the socio-historical context of the genesis of Celan’s poem “Todesfuge” (death fugue) or not knowing his name surely will read and feel about it differently from those whose apperceptive mass includes this information. If the situation described in and providing background for a text is entirely unfamiliar, an appropriate apperceptive mass is lacking. Since there is no way to relate the new information to existing knowledge structures, no interest is generated and little learning can occur. This learning aspect is an important lacuna of the current NCPM, but as Kintsch (1980) rightly pointed out, reading something, or listening to someone speak, are special kinds of learning experiences. Thus a revised version of the NCPM, or upcoming alternative models, should include hypotheses about how learning-during-reading changes the supposed effects of BG and FG features on variables representative for the three levels of processing. Dixon et al.’s (1993) rereading paradigm is an interesting option in this respect (cf. Van Peer, 2007).

A second limitation is the model’s strong focus on “online-aspects” of literary reading. It only considers the (processing of) *microstructural* aspects, i.e., short intervals of reading sections that last from fractions of a second (i.e., single word recognition) to a few minutes and thus loosely lie within the capacity of verbal working memory. Other *macroscopic* aspects such as the structure of a tale or the connections between episodes of a novel which can concern hour- or day-long reading acts are left out, much as processes which precede or follow reading (Mar et al., 2011). According to Wallot (2014), however, very different reading dynamics might be at play when considering long-lasting reading acts. At present, however, extending the NCPM or creating a new model for these aspects seems quite futile given the extreme scarcity of data that could inform such an enterprise. Thus, the primary challenge here is to motivate empirical studies of more natural and ecologically valid reading acts (e.g., Radach et al., 2008; Wallot et al., 2013; Wallot, 2014). An interesting side, or perhaps even, central aspect of such studies could be the question to what extent the processes hypothesized in the NCPM are altered by reading habits and medium, e.g., reading in a (printed) book vs. reading on screens or other digital devices (Mangen et al., 2013). After all, the world of reading (e-)books is changing quickly and drastically, and how these changes alter our reading acts, and thus, our mental life and personalities should be a big issue for research on neurocognitive poetics, too.

## Conflict of Interest Statement

The author declares that the research was conducted in the absence of any commercial or financial relationships that could be construed as a potential conflict of interest.

## Footnotes

^1^Stockwell characterizes Cognitive Poetics as “the application of cognitive science to illuminate the study of literary reading” and “the latest development in the progressive evolution of stylistics”.

^2^Without cross-validation by more implicit and objective measures, the results of such explicit methods may contain confounds with effects of social desirability, personal theories, or non-authentic answer elements. Moreover, not all parts of affective or cognitive responses become conscious and are verbally reportable.

^3^Note that features must not be independent, e.g., iconicity or global affective meaning link phonology with semantics, just as different levels may interact, e.g., the sublexical and supralexical ones (i.e., phoneme salience affecting global affective meaning).

^4^Cited from: Richard L. W. Clarke: http://www.rlwclarke.net; LITS3303 Notes 10B.

^5^English translation: (It was pain beyond anything Harry had ever experienced; his very bones were on fire; his head was surely splitting along his scar; his eyes were rolling madly in his head.)

^6^In a review of research on flow in mediated environments, Novak et al. (2000) identify at least 13 separate constructs used to study flow, with different combinations of these constructs appearing in different investigations.

^7^During fMRI scanning, subjects did not explicitly evaluate the stimuli.

^8^At the time most of the now published studies discussed above were not available.

^9^“Boxological models” being prequantitative, falsification in the strict sense—e.g., as in falsifying Fechner’s law, is not possible. Rather, since they have mainly heuristic value, ‘falsification’ would seriously question or deny exactly this heuristic value.

^10^The model would predict that in the case of an aesthetic appreciation, the above mentioned reward circuits would be active, while in the case of a disgust reaction, the anterior insula would show significant activity, but current technology hardly allows to verify this during on-line reading acts.
